# Atorvastatin Induces Bioenergetic Impairment and Oxidative Stress Through Reverse Electron Transport

**DOI:** 10.3390/antiox14101147

**Published:** 2025-09-23

**Authors:** Francesca Valenti, Luca Pincigher, Nicola Rizzardi, Francesca Orsini, Christian Bergamini, Romana Fato

**Affiliations:** 1 Department of Pharmacy and Biotechnology, FABiT, University of Bologna, Via Irnerio 48, 40126 Bologna, Italy; francesca.valenti8@unibo.it (F.V.); luca.pincigher2@unibo.it (L.P.); nicola.rizzardi2@unibo.it (N.R.); romana.fato@unibo.it (R.F.); 2 Indena SpA, Viale Ortles, 20139 Milan, Italy; francesca.orsini@indena.com

**Keywords:** statin, Coenzyme Q_10_, mitochondria, RET, Ubiqsome

## Abstract

Statins are the first-line therapy for managing elevated cholesterol levels that represent a risk of acute cardiovascular events. However, the use of statins is associated with several side effects, likely due to the depletion of Coenzyme Q_10_ (CoQ_10_), a key component of the mitochondrial electron transport chain and a membrane antioxidant. In our study, we present evidence of the cytotoxic effects of Atorvastatin on human dermal fibroblasts in terms of oxidative stress and mitochondrial impairment. Interestingly, CoQ_10_ supplementation in statin-treated cells significantly reduced ROS levels and restored mitochondrial oxygen consumption rate and the intracellular ATP/ADP ratio. Moreover, our data suggest that the mechanism for Atorvastatin off-target effects at high concentrations involves the inhibition of respiratory complexes I and III, leading to reverse electron transport and ROS production by Complex I. These findings highlight the potential benefits of CoQ_10_ supplementation in mitigating statin-induced cytotoxicity and propose a mechanistic basis for the adverse effects associated with Atorvastatin therapy.

## 1. Introduction

Statins are a class of drugs widely used to treat hypercholesterolemia [1]. Their primary mechanism of action involves the competitive inhibition of 3-hydroxy-3-methyl-glutaryl-CoA reductase (HMG-CoA reductase), the rate-limiting enzyme in the mevalonate pathway, the metabolic pathway that produces cholesterol, Coenzyme Q_10_ (CoQ_10_), and other isoprenoids [2]. In vivo, statins effectively reduce the biosynthesis of cholesterol by their action on the target enzyme and their increasing LDL-cholesterol uptake from the bloodstream by upregulating LDL receptor expression in hepatocytes [3]. The reduction in circulating LDL-cholesterol is crucial in lowering the risk of cardiovascular acute events, such as heart attacks and strokes, making statin therapy one of the most widely used strategies for managing hypercholesterolemia and related cardiovascular diseases [4]. Although generally well tolerated, the long-term use of statins is associated with various side effects that significantly reduce patients’ compliance [5,6]. Muscle-related side effects, such as muscle pain (myalgia) and weakness, are frequently reported. In rare cases, muscle symptoms can progress to rhabdomyolysis, a severe condition characterized by the breakdown of muscle tissue, which can lead to kidney failure [7]. The exact cause of statin-induced myalgia remains unclear; however, various studies have pointed to a potential role of reduced levels of muscle Coenzyme Q_10_ due to mevalonate pathway inhibition, with consequently impaired mitochondrial function. Coenzyme Q_10_ (also known as ubiquinone) is a vitamin-like compound that is obtained partly from the diet, but is primarily biosynthesized via the mevalonate pathway [8]. It is an essential electron carrier in the mitochondrial electron transport chain (ETC), playing a crucial role in energy production, and it is also a potent antioxidant in cell membranes [9]. A deficiency of CoQ_10_ may therefore compromise the mitochondrial respiratory function and increase the generation of reactive oxygen species (ROS). This proposed link between statin therapy, reduced muscle CoQ_10_ levels, mitochondrial dysfunction, and the development of myalgia is biologically plausible, although it has yet to be definitively established in scientific literature, as data obtained on intramuscular CoQ_10_ levels in patients with statin-associated myopathy are scarce [9]. Several studies have investigated the potential benefits of CoQ_10_ supplementation in alleviating the adverse effects of statins. However, the evidence of the benefits of CoQ_10_ supplementation in vivo is inconclusive, with some studies finding no consistent improvement in symptoms among statin-treated patients [10,11,12].

One of the challenges associated with CoQ_10_ supplementation is represented by its poor bioavailability, due to its high molecular weight and poor water solubility. Various formulations have been developed to overcome this issue. In this study, we used UBIQSOME^®^, a formulation in which CoQ_10_ is carried by lecithin-based phospholipids (phytosome). UBIQSOME^®^ has already proved to be effective and well uptaken in both in vitro and in vivo models [13,14]. Using UBIQSOME^®^, we demonstrate that CoQ_10_ supplementation can effectively reverse the mitochondrial dysfunction, bioenergetic imbalance, and oxidative stress induced by Atorvastatin treatment in human dermal fibroblasts (HDF).

In addition, statin accumulation due to prolonged treatment, high dosage, or impaired clearance can lead to off-target effects, both in vivo and in vitro, which extend beyond a decrease in CoQ_10_ levels [15,16]. We investigated the effect of high doses of Atorvastatin on the mitochondrial respiratory chain enzymes, finding a direct inhibition of Complexes I and III along with evidence suggesting an Atorvastatin-induced reverse electron transport (RET) with ROS production by Complex I. This could be an important mechanism underlying the side effects of statin use and could be of clinical interest for developing strategies to mitigate these adverse effects, such as ubiquinone supplementation. While these in vitro findings provide valuable mechanistic insights into the molecular basis of statin-induced side effects, further studies are needed to validate the results in vivo.

## 2. Materials and Methods

### 2.1. Cell Culture and Treatment

Human dermal fibroblasts (HDF) from a healthy middle-aged donor were cultured in Dulbecco’s Modified Eagle Medium (DMEM), supplemented with 10% fetal bovine serum (FBS), 100 I.U./mL Penicillin, and 100 μg/mL Streptomycin, and grown at 37 °C in 5% CO_2_ with saturating humidity. For drug treatment, cells were seeded on an appropriate support and grown for 24 h in complete culture medium before treatment. Cell count for seeding was performed by using the Trypan blue exclusion method [17]; protein content was assessed by using the Lowry method [18]. Treatments were performed using Atorvastatin Calcium, UBIQSOME^®^ (UBQ) or a combination of the two. Atorvastatin Calcium was purchased at Sigma-Aldrich (St. Louis, MO, USA) and dissolved in bi-distilled water-prepared 10% Bovine Serum Albumin (BSA, Sigma-Aldrich, St. Louis, MO, USA) for cell treatments or DMSO for enzymatic assays; UBQ was provided by Indena S.p.A., Milan, Italy; concentrations of UBQ used refer to the equivalent CoQ_10_ present in the formulation.

### 2.2. Viability Assay

First, 3 × 10^3^ HDF per well were seeded on a multi-96 well plate and allowed to attach for 24 h before treatment. For the treatments, cells were incubated with a range of concentrations between 3 μM and 100 μM of Atorvastatin for 24 h and co-treated with 10 nM, 50 nM, or 100 nM of UBQ. For the cell viability analysis, cells were subjected to the MTT colorimetric assay [19]. Briefly, cells were incubated with 300 μM MTT dissolved in non-complete DMEM for 2 h at 37 °C; after this time, medium was aspirated, the wells were then gently washed with PBS, and 150 μL of DMSO per well was added to dissolve formazan salts. Absorbance was read at λ = 570 nm using a Victor Nivo multiplate reader spectrophotometer (PerkinElmer, Waltham, MA, USA).

### 2.3. Oxygen Consumption Rate

The oxygen consumption rate was measured in HDF using a Seahorse XF24 Extracellular Flux Analyzer (Agilent, Santa Clara, CA, USA). Next, 1 × 10^4^ cells per well were seeded onto a Seahorse XF24 cell culture microplate and incubated for 24 h prior to treatment with 30 μM, 50 μM, and 100 μM Atorvastatin with and without 10 nM UBQ. Plates were washed with Seahorse assay media (Seahorse Bioscience, Billerica, MA, USA), supplemented with 1 g/L glucose, 1 mM sodium pyruvate, and 2 mM L-glutamine, and incubated in a CO_2_-free incubator at 37 °C for 1 h to allow temperature and pH equilibration before measuring in the Seahorse XF24. OCR was recorded in basal conditions and after the addition of inhibitors and effectors of the mitochondrial electron transport chain: oligomycin 0.4 μM to inhibit ATP synthase; FCCP (Carbonyl cyanide 4-(trifluoromethoxy) Phenylhydrazone) 2 μM to uncouple respiration; and rotenone 1 μM and antimycin A 5 μM to block the mitochondrial chain at the end of the experiment. Cells were counted using the Cell Imaging Reader BioTek Cytation 1 (Agilent, Santa Clara, CA, USA) prior to the analysis to normalize data.

### 2.4. Bioenergetic Status

Nucleotides were extracted following Jones et al. [20] with minor modifications and quantified by HPLC. Briefly, 1.5 × 10^5^ cells were seeded in a T25 flask and allowed to adhere for 24 h before treatments. Extracted nucleotides were injected in a two-pump system equipped with a photodiode array detector (Agilent, Santa Clara, CA, USA, 1100 series) and a C18 column (Kinetex, Phenomenex, Torrance, CA, USA, 2.6 μm, 250 mm × 4.6 mm) with λ = 260 nm. ATP and ADP were quantified by measuring the area under the curve of identified peaks and interpolating the result in a standard curve. Data were then normalized on protein content.

### 2.5. Coenzyme Q_10_ Content

CoQ_10_ was extracted from cultured cells as described by Takada et al. [21] with minor modifications. Briefly, cells were pelleted and resuspended in 200 μL PBS. A total of 10 μL of internal standard CoQ_7_, 10 μL of FeCl_3_ 0.1%, and 5 volumes of a n-hexane/ethanol mixture (5:3) were added. The suspension was thoroughly vortexed for 2 min and centrifuged at 1800× *g* for 10 min. The upper layer from each sample was collected, and a second extraction was performed. The collected solutions were dried out in glass tubes by nitrogen flux, and the dry extracts were resuspended in 50 μL of ethanol. Next, 20 μL of samples were injected into a two-pump HPLC system equipped with photodiode array detector (Agilent, Santa Clara, CA, USA) and a C18 column (Kinetex, Phenomenex, 5 μm 100 A, 150 × 4.6 mm), using an ethanol/water mobile phase (96:4, *v*/*v*) at a 0.8 mL/min flow rate. The CoQ_7_ and CoQ_10_ peaks at λ = 275 nm were identified and quantified by interpolating the area under the curve with a calibration curve. The results were normalized on protein content and extracted CoQ_7_.

### 2.6. Mitochondrial Mass

Mitochondrial mass was assessed by measuring the citrate synthase activity [22] in a Jasco V-750 spectrofluorometer (Jasco, Tokyo, Japan) equipped with a stirring device and thermostatic control set up at 30 °C. Briefly, 30 μg of cell lysate were added to a 1 mL quartz cuvette containing a 100 mM TRIS + 0.1% Triton X-100 buffer (pH 7.4), 100 μM of acetyl-CoA, and 100 μM of 5,5′-dithiobis-2-nitrobenzoic acid (DTNB), and 500 μM oxalacetate was used to start the reaction. The enzymatic activity was measured following the reduction of DTNB to TNB (ε = 13.6 mM^−1^ cm^−1^) at λ = 412 nm over time.

### 2.7. Mitochondrial Chain Functionality in Permeabilized Cells

The activity of the electron transport chain (ETC) was measured by OCR using a Seahorse XF24 Extracellular Flux Analyzer (Agilent, Santa Clara, CA, USA) after 24 h treatment with 30 μM Atorvastatin, 10 nM UBQ, or both. A total of 1.5 × 10^4^ cells were seeded in a Seahorse XF24 cell culture microplate and incubated for 24 h prior to treatment; the day of the experiment the plates were washed with Seahorse assay media (Seahorse Bioscience) and incubated in a CO_2_-free incubator at 37 °C for 1 h to allow temperature and pH equilibration before measuring in the Seahorse XF24. Cells were permeabilized with digitonin (25 μg/mL) and supplemented with substrates for Complex I or Complex II. Namely, to measure Complex I-driven OCR, cells were incubated with 2.5 mM glutamate/malate and 40 μM malonate, while Complex II-related respiration was conducted by adding 10 mM succinate and 1 μM rotenone to the medium. Basal oxygen consumption was measured.

### 2.8. Mitochondrial Chain Functionality in Isolated Mitochondria

The activity of the mitochondrial chain was measured in isolated freeze–thawed mouse liver mitochondria. Mouse liver mitochondria were isolated according to Bergamini et al. [23] with minor modification; to permeabilize the membranes and provide substrates, isolated mitochondria were subjected to freeze–thaw cycles. Complex I-driven respiration was measured in a Jasco V-750 spectrofluorometer (Jasco, Tokyo, Japan) equipped with a stirring device and thermostatic control set up at 30 °C. A total of 50 μg of mitochondria were incubated with different concentrations of DMSO-dissolved Atorvastatin and diluted in a 1 mL solution containing 25 mM potassium phosphate buffer pH 7.5 and 1 mg/mL BSA in a quartz cuvette. The reaction was initiated by adding 70 μM of NADH. The enzymatic activity was measured following the extinction of NADH at λ = 340 nm over time. Complex II-driven respiration was conducted in an oxygraphy chamber (Instech Mod.203, Plymouth Meeting, PA, USA). Briefly, 275 μg of mitochondria were added to the oxygraphy chamber, which contained a respiration buffer (0.25 M sucrose, 50 mM HEPES, 4 mM MgSO_4_, 10 mM KH_2_PO_4_, pH 7.4). The reaction was started by adding 20 mM of succinate to the chamber.

### 2.9. Specific Complex I Activity

Specific Complex I hydrogenase activity in the presence of Atorvastatin was assayed spectrophotometrically, as in Spinazzi et al. [24], using a Jasco V-750 spectrofluorometer (Jasco, Tokyo, Japan) equipped with a stirring device at 30 °C. Briefly, 30 μg of isolated freeze–thawed mouse liver mitochondria were pre-incubated with Atorvastatin and then transferred in a 1 mL quartz cuvette containing 25 μM antimycin A, 1 mg/mL BSA and 70 μM NADH (ε = 6.22 mM^−1^ cm^−1^) dissolved in a 50 mM potassium phosphate buffer; 50 μM decyl-ubiquinone (DB) was used to start the reaction and NADH consumption was followed over time at λ = 340 nm. NADH dehydrogenase activity of Complex I was measured by following ferricyanide [Fe(CN)_6_]^3−^ (ε = 1 mM^−1^ cm^−1^) reduction [25] in a Jasco V-750 spectrofluorometer (Jasco, Tokyo, Japan) equipped with a stirring device at 30 °C. Briefly, 30 μg of isolated freeze–thawed mouse liver mitochondria were pre-incubated with Atorvastatin and then transferred in a 1 mL quartz cuvette containing a 50 mM potassium phosphate buffer, 1 mg/mL BSA and 25 μM antimycin A; 70 μM NADH and 250 μM of [Fe(CN)_6_]^3−^ were used to start the reaction and [Fe(CN)_6_]^3−^ reduction was followed over time at λ = 420 nm.

### 2.10. Specific Complex II Activity

Complex II activity in the presence of Atorvastatin was assayed spectrophotometrically, as in Spinazzi et al. [24], using a Jasco FP-770 spectrofluorometer (Jasco, Tokyo, Japan) equipped with a stirring device at 30 °C. Briefly, isolated freeze–thawed mouse liver mitochondria were pre-incubated with Atorvastatin and then transferred in a 1 mL quartz cuvette containing 25 μM antimycin and 20 mM succinate dissolved in a potassium phosphate buffer at pH 7.5; 50 μM decyl-ubiquinone (DB) and 2,6-Dichlorophenolindophenol sodium salt hydrate (DCPIP, ε = 16 mM^−1^ cm^−1^) were used to start the reaction; DCPIP reduction was followed over time at λ = 600 nm.

### 2.11. Measurement of Cytosolic ROS Production

Reactive oxygen species (ROS) production was measured in cultured HDF using the chloromethyl derivative of 2′-7′-dichlorodihydrofluorescein diacetate, acetyl ester (CM-H_2_DCFDA, Thermo Fisher Scientific, Waltham, MA, USA). A total of 2 × 10^4^ cells per well were seeded onto a 24-well plate and incubated for 24 h with 30 μM of Atorvastatin, 10 nM UBQ, or both. Positive controls were generated by adding 300 μM of hydrogen peroxide. Then, cells were loaded with 5 μM of the probe for 45 min at 37 °C and washed twice with Hanks’ Balanced Salt Solution (HBSS). Epifluorescence was captured using a Zeiss Celldiscoverer7 (Zeiss, Jena, Germany), in a controlled atmosphere (37 °C, 5% CO_2_), with the green channel. Single-cell fluorescence intensity was analyzed with ImageJ Version 1.54p.

### 2.12. Measurement of Mitochondrial ROS Production in Intact Cells

Mitochondrial ROS production (mROS) was measured in cultured HDF using the dihydroethidium derivative MitoSOX Red or MitoSOX Green (Thermo Fisher, Waltham, MA, USA). For MitoSOX Red staining, 1 × 10^4^ cells per well were seeded in a μ-Slide 8 Well (Ibidi, Germany) and incubated for 24 h prior to treatment with 30 μM Atorvastatin or 10 nM UBQ. For mROS detection cells were loaded with 5 μM MitoSOX Red for 30 min at 37 °C and washed twice with HBSS before measurement. The cells were then imaged using a Leica SPE confocal microscope (Leica Microsystems, Wetzlar, Germany) and analyzed using ImageJ software Version 1.54p (National Institutes of Health, Bethesda, MD, USA). For MitoSOX Green staining, 3 × 10^4^ cells per well were seeded in a multi-96 plate and incubated for 24 h prior to treatment with 30 μM Atorvastatin. Cells were stained with 1 μM MitoSOX Green for 30 min at 37 °C and washed twice with HBSS; 20 mM succinate in HBSS was added to each well before measurement. Green fluorescence was acquired using a multi-plate reader spectrophotometer (PerkinElmer Victor Nivo, Waltham, MA, USA).

### 2.13. Measurement of Membranes Peroxidation

The determination of membrane lipid peroxidation was performed using the lipid peroxidation sensor dye STY-BODIPY [26]. A total of 1 × 10^4^ cells per well were seeded in a μ-Slide 8 Well (Ibidi, Germany) and incubated for 24 h prior to treatment with 30 μM Atorvastatin or UBQ. The day of the experiment, cells were incubated with 1 μM of the probe for 1 h, washed with PBS, and fixed in 4% paraformaldehyde. Positive controls were generated using 500 nM of RSL3, an inducer of lipid peroxidation. Images were acquired using a Nikon C1si confocal microscope (Nikon, Tokyo, Japan) and fluorescence intensities were quantified using ImageJ software Version 1.54p. Fluorescence was acquired by two-channel imaging, as the probe emission peak shifts from λ = ~590 nm to λ = ~510 nm when oxidized. Data are reported as green/red fluorescence intensity ratio.

### 2.14. ROS Production in Isolated Mitochondria

ROS production in isolated mouse liver mitochondria was followed over time as in Fato et al. [27] with minor modifications. A total of 0.5 mg/mL mitochondria were energized with 70 μM NADH, or 20 mM succinate, in the presence of different concentrations of DMSO-dissolved Atorvastatin ranging from 12.5 μM to 100 μM, and 5 μM of the fluorogenic dye 2′,7′-Dichlorodihydrofluorescein diacetate (DCFDA). The fluorescence of the oxidized DCF was recorded every 5 min in a multi-plate reader spectrophotometer (PerkinElmer Victor Nivo, Waltham, MA, USA).

### 2.15. Statistical Analysis

The statistical analysis was performed using GraphPad Prism (Version 8, San Diego, CA, USA). The values are expressed as means ± standard error of the mean (SEM). The significance of the results was obtained using the Brown–Forsythe ANOVA test. A probability level of *p* < 0.05 was considered to be statistically significant.

## 3. Results

### 3.1. Atorvastatin Reduces Cell Viability

**Figure 1:**
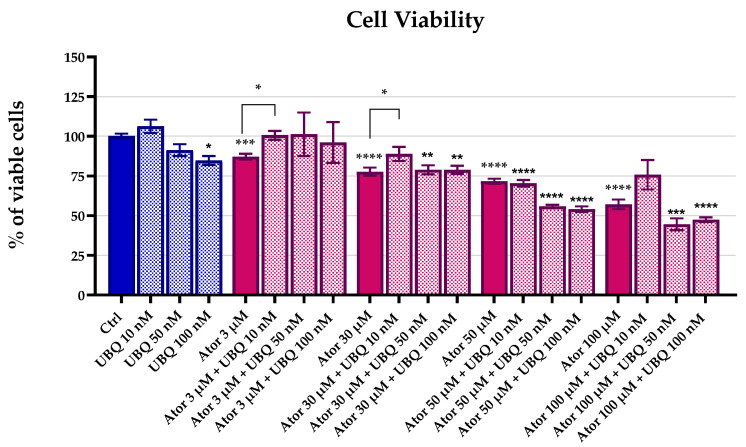
Cell viability of human dermal fibroblasts following 24 h treatment with Atorvastatin (3, 30, 50, or 100 μM) alone or in combination with UBIQSOME^®^ (10, 50, or 100 nM), as determined by MTT assay. Data represent the percentage of viable cells relative to untreated controls and are expressed as mean ± SEM (*n* = 5). Statistical significance was assessed using Brown–Forsythe ANOVA (* *p* ≤ 0.05; ** *p* ≤ 0.01; *** *p* ≤ 0.001; **** *p* ≤ 0.0001). Abbreviations: Ator = Atorvastatin; UBQ = UBIQSOME^®^.

We investigated the effect of Atorvastatin treatment on HDF viability using the MTT test. Cells were incubated with 3 μM, 30 μM, 50 μM, and 100 μM of Atorvastatin in the presence or absence of different concentrations of UBQ for 24 h. The MTT test revealed a dose-dependent reduction in cell viability after Atorvastatin treatment. The UBQ supplementation was partially able to rescue cell viability at 10 nM. (Figure 1).

### 3.2. Atorvastatin Induces Bioenergetic Impairments

**Figure 2:**
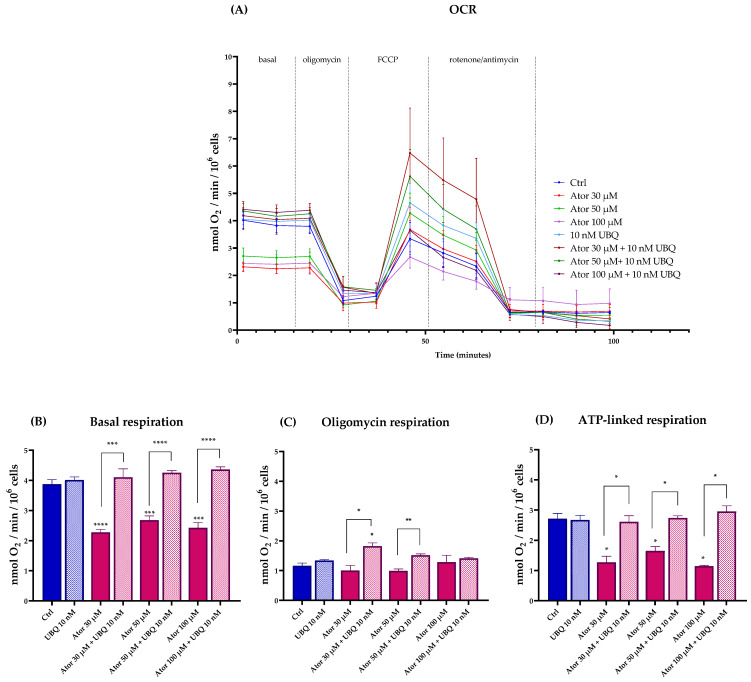
Oxygen consumption rate in cells treated with various concentrations of Atorvastatin with and without supplementation with 10 nM UBQ for 24 h. (**A**) Representative OCR profile, (**B**) Basal respiration, (**C**) respiration in the presence of oligomycin, (**D**) ATP-linked respiration calculated as the difference between basal respiration and oligomycin-respiration. Data are mean ± SEM (*n* = 3). Statistical significance was assessed using Brown–Forsythe ANOVA (* *p* ≤ 0.05; ** *p* ≤ 0.01; *** *p* ≤ 0.001; **** *p* ≤ 0.0001). Abbreviations: Ator = Atorvastatin; UBQ = UBIQSOME^®^.

We investigated the effect of Atorvastatin on mitochondrial functionality by measuring the oxygen consumption rate (OCR, Figure 2A) in fibroblasts using the Seahorse XF24 Extracellular Flux Analyzer (Agilent). The basal and ATP-linked respirations were significantly reduced by 30, 50, and 100 μM Atorvastatin treatment, leaving oligomycin-related respirations unaltered; co-supplementation with 10 nM of UBQ was able to restore the altered parameters to control levels (Figure 2B–D).

**Figure 3:**
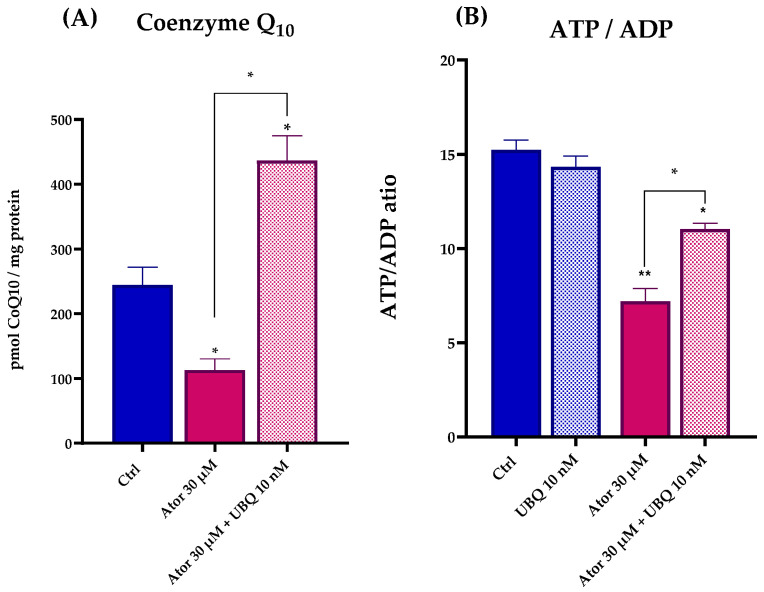
Coenzyme Q_10_ quantitation (**A**) and ATP/ADP ratio (**B**) after 24 h treatment with 30 μM Atorvastatin and 10 nM UBQ. Data are mean ± SEM (*n* = 3). Statistical significance was assessed using Brown–Forsythe ANOVA (* *p* ≤ 0.05; ** *p* ≤ 0.01). Abbreviations: Ator = Atorvastatin; UBQ = UBIQSOME^®^.

We used high-performance liquid chromatography (HPLC) to quantify the intracellular Coenzyme Q_10_ and ATP/ADP levels in HDF treated with 30 μM Atorvastatin for 24 h. Under these conditions, we observed a drastic drop in the biosynthesis of CoQ_10_, and a lower ATP/ADP ratio, indicating severe bioenergetic impairment. Consistent with the respiratory data, co-treatment with 10 nM UBQ restored CoQ_10_ levels and the cellular energy deficiency was partially recovered (Figure 3A,B).

**Figure 4:**
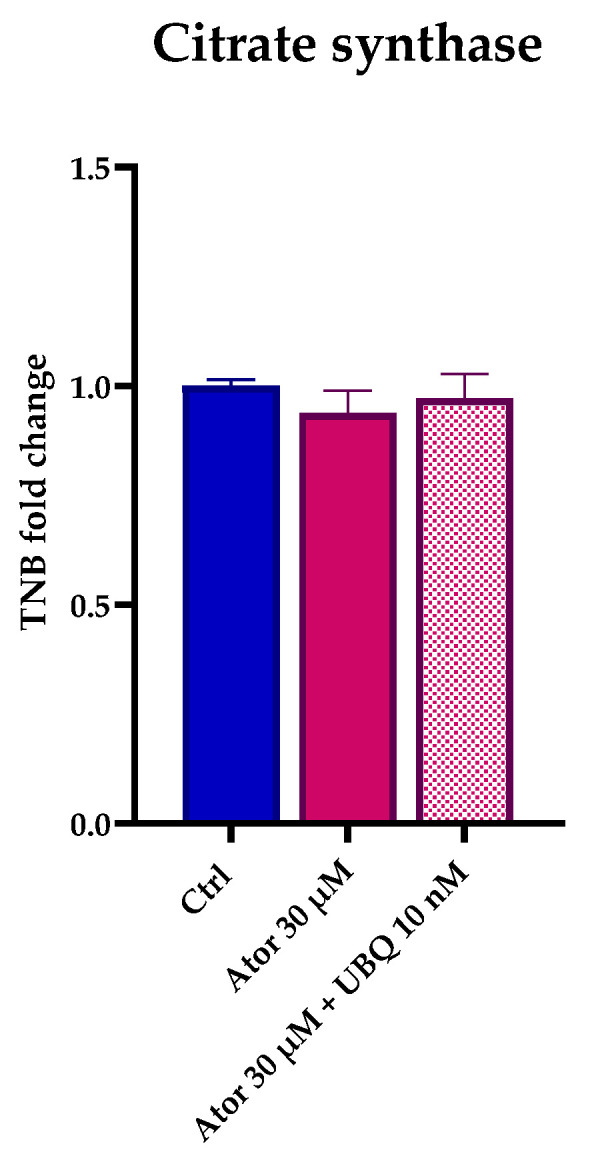
Mitochondrial mass after 24 h treatment with 30 μM Atorvastatin with and without 10 nM UBQ detected as amount of TNB. Data are mean ± SEM (*n* = 3). Statistical significance was assessed using Brown–Forsythe ANOVA. Abbreviations: Ator = Atorvastatin; UBQ = UBIQSOME^®^.

Citrate synthase enzymatic activity is considered a reliable marker by which to estimate mitochondrial content. Despite the observed impairment of the mitochondrial function, the overall mitochondrial mass was not altered after treatment with Atorvastatin, as indicated by the unaltered citrate synthase activity (Figure 4). Moreover, RT-PCR analysis confirmed that the expression of CS and other genes central to mitochondrial function (namely SDH, PGC1α and ETFDH) were not significantly altered in HDF after Atorvastatin treatment (Figure S1).

#### 3.2.1. Atorvastatin Impairs the Mitochondrial Electron Transport Chain in Permeabilized HDF

**Figure 5:**
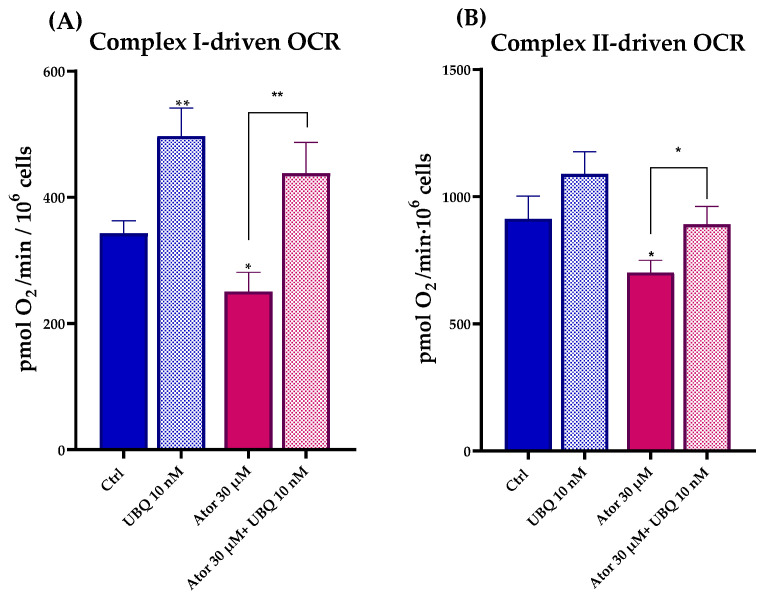
Mitochondrial respiratory activity in permeabilized human dermal fibroblasts following 24 h treatment with 30 μM Atorvastatin alone or in combination with 10 nM UBQ. (**A**) Complex I-driven and (**B**) Complex II-driven oxygen consumption rates were measured by high-resolution respirometry. Data are expressed as mean ± SEM (*n* = 3). Statistical significance was assessed using Brown–Forsythe ANOVA (* *p* ≤ 0.05; ** *p* ≤ 0.01). Abbreviations: Ator = Atorvastatin; UBQ = UBIQSOME^®^.

To study the effect of Atorvastatin on the ETC, we measured Complex I- and Complex II-driven respirations in digitonin-permeabilized HDF after treatment with 30 μM Atorvastatin for 24 h using the Seahorse XF24 Extracellular Flux Analyzer. Complex I-dependent OCR was measured in the presence of glutamate and malate, inhibiting Complex II with malonate; Complex II-dependent respiration was assessed after the addition of succinate, inhibiting Complex I with rotenone. In both cases, Atorvastatin-treated cells showed a decrease in basal respiration, which could be due to the decrease in endogenous CoQ_10_ levels. Co-treatment with 10 nM UBQ and 30 μM Atorvastatin recovers the oxygen consumption in the presence of both glutamate/malate and succinate (Figure 5A,B).

#### 3.2.2. Atorvastatin Directly Impairs the Mitochondrial Electron Transport Chain Activity in Isolated Mitochondria

**Figure 6:**
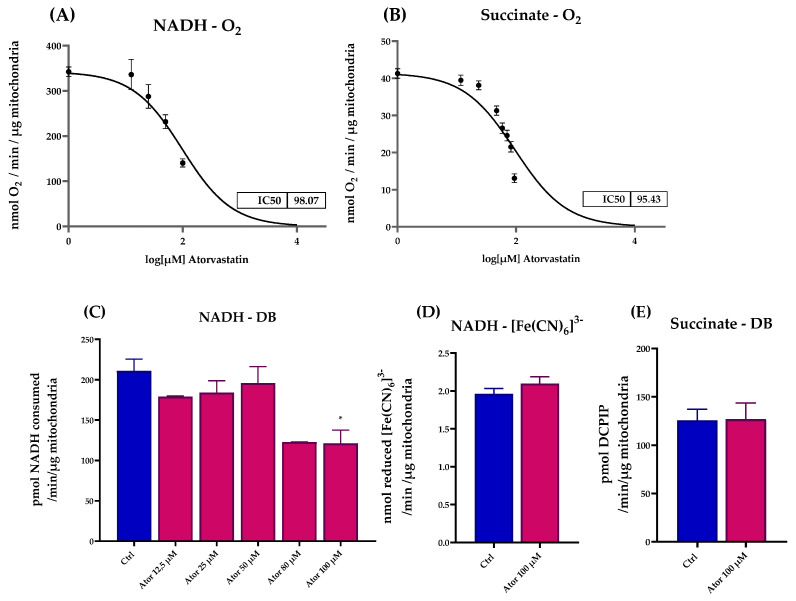
Dose-dependent effects of Atorvastatin on mitochondrial respiratory chain function in isolated mouse liver mitochondria. (**A**) NADH–O_2_ activity measured at various concentrations of Atorvastatin. NADH oxidation was monitored spectrophotometrically at λ = 340 nm. (**B**) Succinate–O_2_ activity in the presence of different concentrations of Atorvastatin, assessed by oxygen consumption using a Clark-type oxygen electrode. (**C**) Functionally isolated Complex I activity in the presence of different concentrations of Atorvastatin in mitochondria energized with NADH and using decyl-ubiquinone (DB) or (**D**) ferricyanide ([Fe(CN)_6_]^3−^) as electron acceptors. NADH oxidation and [Fe(CN)_6_]^3−^ reduction were monitored spectrophotometrically at λ = 340 nm and λ = 420 nm, respectively. (**E**) Functionally isolated Complex II activity measured in the presence of different concentrations of Atorvastatin. Mitochondria were energized with succinate and the reduction in the electron acceptor DCPIP (in the presence of DB) was followed at λ = 600 nm. Data are presented as mean ± SEM. Statistical analysis was performed using the Brown–Forsythe ANOVA test (* *p* ≤ 0.05). Abbreviations: Ator = Atorvastatin; DB = decyl-ubiquinone; [Fe(CN)_6_]^3−^ = ferricyanide.

We assessed the effect of Atorvastatin treatment on ETC activity in isolated mouse liver mitochondria, finding a dose-dependent inhibition of both NADH-O_2_ and Succinate-O_2_ activities with similar IC50 value (98.07 μM and 95.43 μM, respectively), as reported in Figure 6A,B. To identify which ETC enzyme might be directly affected by Atorvastatin, we tested the activity of functionally isolated Complex I using either decyl-ubiquinone (DB), a CoQ_10_ analog, or ferricyanide ([Fe(CN)_6_]^3−^) as electron acceptors. Atorvastatin inhibited the NADH-DB reductase activity, while rotenone insensitive NADH-[Fe(CN)_6_]^3−^ activity remained unaffected (Figure 6C,D). Notably, the activity of functionally isolated Complex II, tested in the presence of succinate, DB, and DCPIP as electron acceptor, was not affected by Atorvastatin treatment. (Figure 6E).

### 3.3. Effect of Atorvastatin on ROS Levels in HDF and Isolated Mitochondria

**Figure 7:**
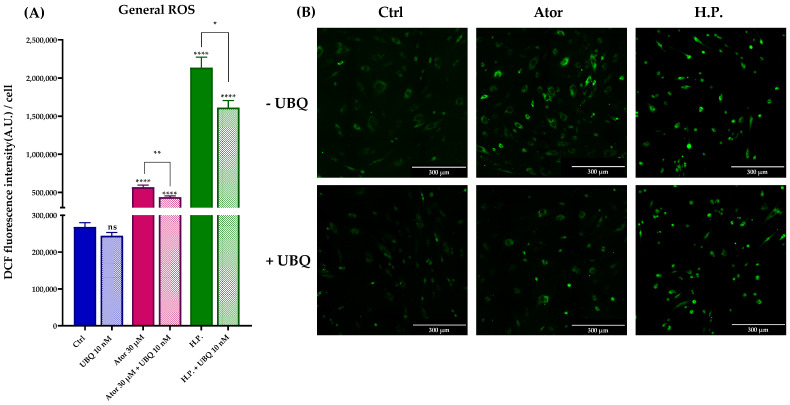
Evaluation of oxidative stress in HDF after treatment with 30 μM Atorvastatin and supplementation with 10 nM UBQ. (**A**) General ROS detected by means of DCF epifluorescence and (**B**) relative images acquired by widefield fluorescence (Scale bar: 300 μm). Data are mean ± SEM. Statistical analysis was performed using the Brown–Forsythe ANOVA test (* *p* ≤ 0.05; ** *p* ≤ 0.01; **** *p* ≤ 0.0001; ns > 0.05). Abbreviations: Ator = Atorvastatin; UBQ = UBIQSOME^®^; H.P. = Hydrogen Peroxide.

Given the controversial role attributed to statins as pro-oxidant or antioxidant drugs, we tested the ROS production in HDF treated with Atorvastatin. General ROS levels were measured using CM-H_2_DCFDA, mitochondrial superoxide with MitoSOX Red, and lipid peroxidation with the STY-BODIPY probe. HDF treated for 24 h with 30 μM Atorvastatin showed increased DCF signal, while co-treatment with 10 nM UBQ reduced oxidative stress. Moreover, UBQ treatment decreased both the basal level and the hydrogen peroxide-induced oxidative stress, which was used as a positive control (Figure 7A,B).

**Figure 8:**
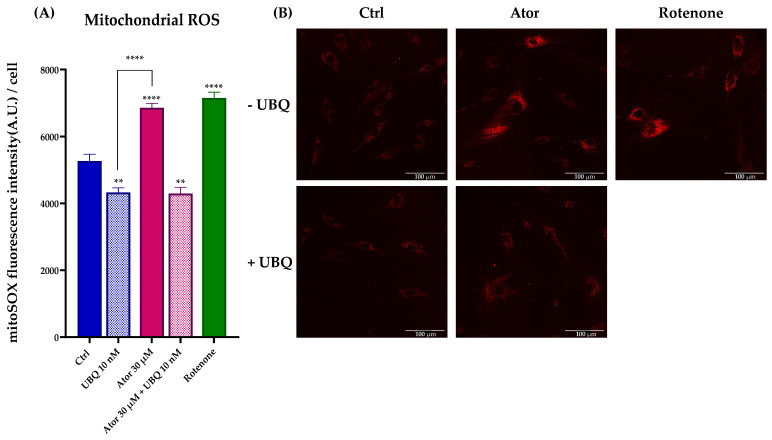
Evaluation of oxidative stress in HDF mitochondria after treatment with 30 μM Atorvastatin and supplementation with 10 nM UBQ. (**A**) Mitochondrial ROS detected with MitoSOX Red by confocal microscopy and (**B**) representative images (Scale bar: 100 μm:). Data are mean ± SEM. Statistical analysis was performed using the Brown–Forsythe ANOVA test (** *p* ≤ 0.01; **** *p* ≤ 0.0001). Abbreviations: Ator = Atorvastatin; UBQ = UBIQSOME^®^.

To investigate the mitochondrial contribution to Atorvastatin-induced ROS, we measured mitochondrial superoxide production using the MitoSOX Red probe in HDF treated with 30 μM Atorvastatin for 24 h; rotenone was used as a positive control. Atorvastatin treatment significantly increased mitochondrial superoxide production, which was diminished by 10 nM UBQ co-treatment (Figure 8A,B).

**Figure 9:**
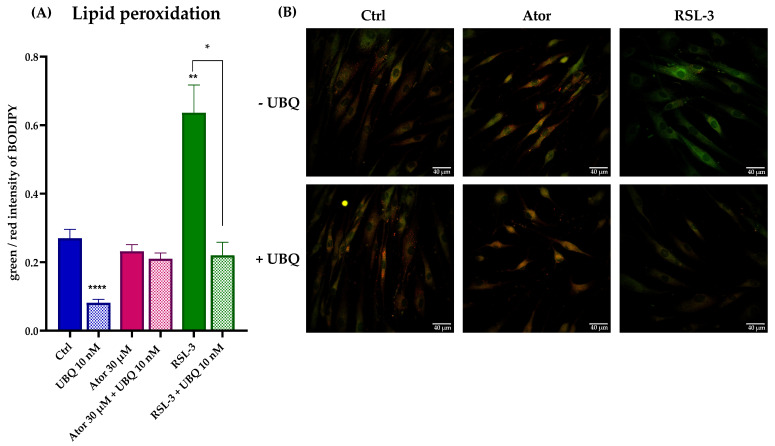
Evaluation of oxidative stress in HDF membranes after treatment with 30 μM Atorvastatin and supplementation with 10 nM UBQ. (**A**) Lipid peroxidation quantitation expressed as green/red ratio of Bodipy fluorescence intensity acquired by confocal microscopy and (**B**) representative images (Scale bar: 40 μm). Data are mean ± SEM. Statistical analysis was performed using the Brown–Forsythe ANOVA test (* *p* ≤ 0.05; ** *p* ≤ 0.01; **** *p* ≤ 0.0001). Abbreviations: Ator = Atorvastatin; UBQ = UBIQSOME^®^.

Lastly, lipid peroxidation was evaluated using the STY-BODIPY dye in cells treated with 30μM Atorvastatin and/or 10 nM UBQ; RSL3, a selective inhibitor of glutathione peroxidase 4 was used as a positive control. We found no discernible effect on the levels of lipid peroxidation induced by Atorvastatin. However, UBQ treatment conferred protection against lipid membrane peroxidation in both control and RSL3-treated cells (Figure 9A,B).

**Figure 10:**
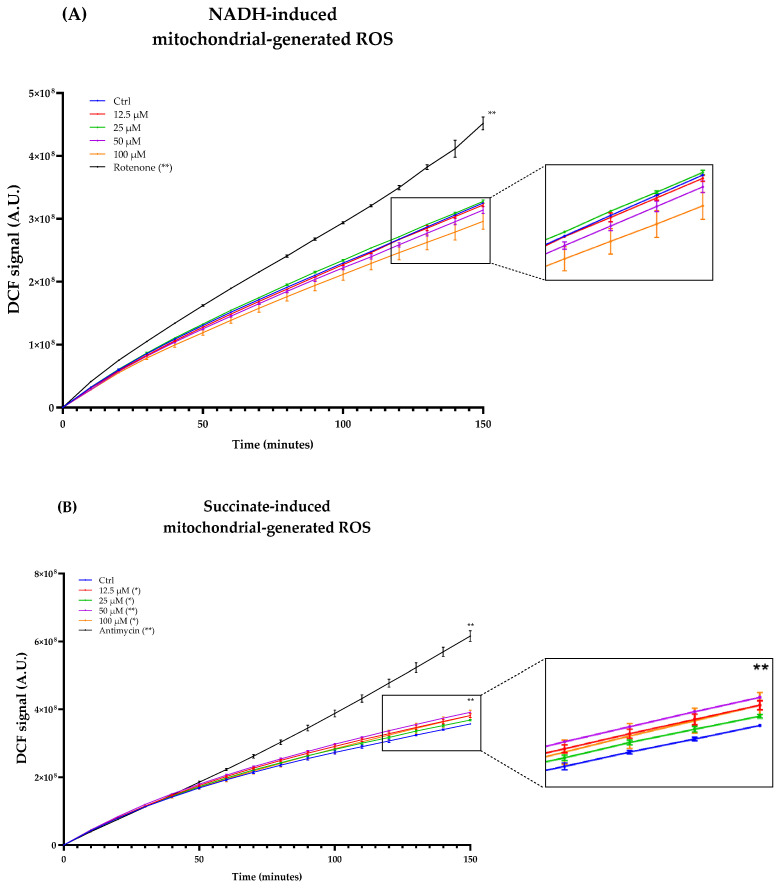
ROS production in isolated mouse liver mitochondria incubated with Atorvastatin. Mitochondria were incubated with 12.5, 25, 50, and 100 μM Atorvastatin and 5 μM DCFDA, energized with NADH (**A**) or succinate (**B**). Fluorescence was detected using a multi-plate reader every 5 min. Data are mean ± SEM. Statistical analysis was performed using the Brown–Forsythe ANOVA test (* *p* ≤ 0.05; ** *p* ≤ 0.01). Abbreviations: Ator = Atorvastatin.

As Complexes I and III of the ETC are considered the major ROS producers, we titrated ROS production in freeze–thawed isolated mouse mitochondria incubated with NADH or succinate in the presence of different concentrations of Atorvastatin. DCF fluorescence showed that Atorvastatin had no effect on ROS production using NADH as an electron donor, while radical species increased slightly using succinate (Figure 10A,B).

**Figure 11:**
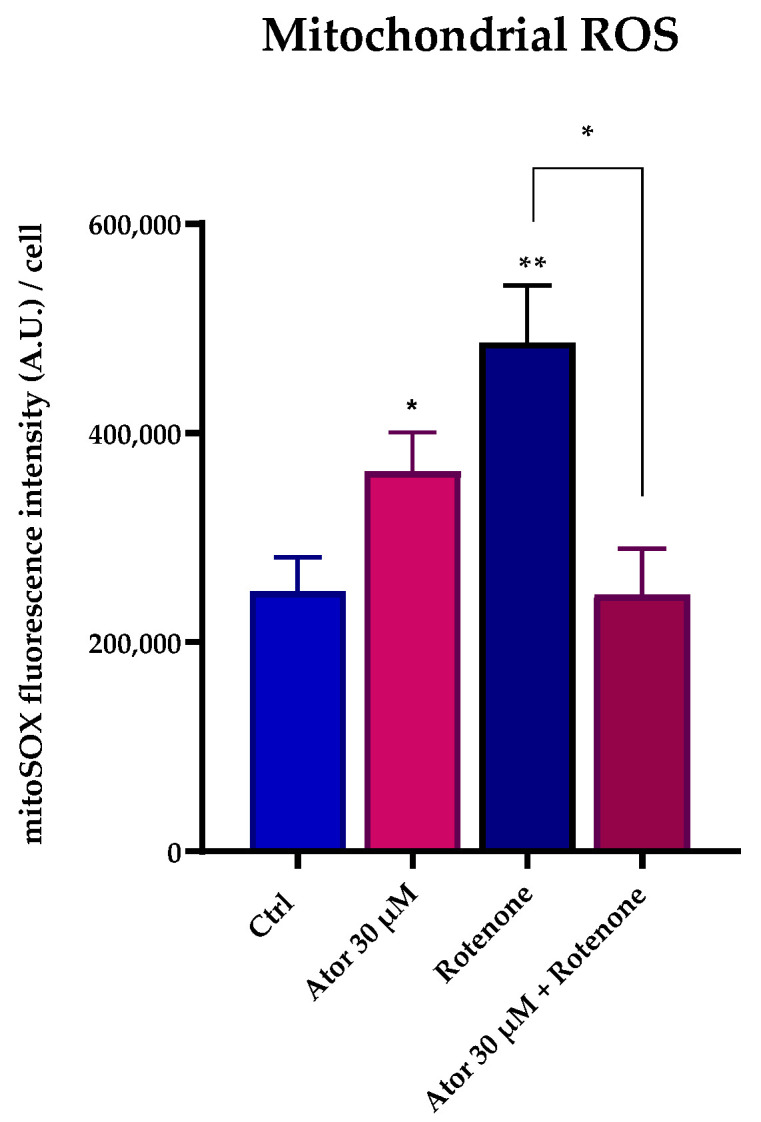
Atorvastatin induces ROS production via Reverse Electron Transport. Quantitation of MitoSOX Green in cells treated with 30 μM Atorvastatin, 5 μM rotenone, or both, and energized with 20 mM succinate. Data are mean ± SEM. Statistical analysis was performed using the Brown–Forsythe ANOVA test (* *p* ≤ 0.05; ** *p* ≤ 0.01). Abbreviations: Ator = Atorvastatin.

Nonetheless, ROS production at the mitochondrial level was observed in intact cells (Figure 8A,B). To address the discrepancy between the increased ROS production in live cells and the minimal ROS production detected in isolated mitochondria following Atorvastatin treatment, we supplemented Atorvastatin-treated HDFs with succinate and assessed mitochondrial superoxide production using the MitoSOX Green probe. As shown in Figure 11, both Atorvastatin and rotenone treatments increased ROS levels. Notably, co-incubation with rotenone and Atorvastatin abolished superoxide generation, suggesting that ROS are generated by Complex I from reverse electron transfer (RET).

## 4. Discussion

Statin therapy is broadly used for both primary and secondary prevention of cardiovascular diseases, as its efficacy in lowering circulating LDL-cholesterol significantly decreases the insurgence of major cardiovascular events and microvascular complications [28]. Despite being considered overall safe, with a benefit/risk ratio that justifies their prescription, statins are associated with multiple side effects, particularly muscle-related symptoms, such as pain and weakness, that undermine the patients’ compliance with therapy. The molecular mechanisms underlying these effects, both in vitro and in vivo, remain to be clarified. Therapy efficiency and side effects can vary significantly between patients [29], and in vitro outcomes are also controversial, likely depending on the cell model used [30,31]. This study provides significant insights into the impact of Atorvastatin, a commonly prescribed statin, on the mitochondrial functions of human dermal fibroblasts (HDF). Atorvastatin-treated HDF displayed impaired OCR, a decrease in ATP/ADP ratio, and increased oxidative stress. It is assumed that statin-related symptoms are caused by a reduction in Coenzyme Q_10_, which is biosynthesized through the mevalonate pathway. We observed a drastic reduction in total CoQ_10_ content after Atorvastatin treatment, but it is not clear whether the bioenergetic impairment is due to CoQ_10_ deficiency in the ETC, because of a lowering of antioxidant defenses, or both. We evaluated the efficacy of UBIQSOME^®^, a formulated CoQ_10_, in alleviating these alterations. Our results demonstrated that co-supplementation of CoQ_10_ in Atorvastatin-treated cells is efficient in recovering the altered parameters in terms of ROS protection, ETC efficiency, OCR sustained by glutamate/malate and succinate, and ATP/ADP ratio. However, seeing the plethora of statin-associated symptoms [32], it would be oversimplifying to state that Atorvastatin-induced side effects rely only on CoQ_10_ deficiency. We observed a direct inhibition on functionally isolated Complex I at the CoQ_10_ binding site level. Complex I can be functionally divided into three modules: the dehydrogenase module, the hydrogenase module, and the proton pump module [33,34]. Atorvastatin inhibits Complex I activity at the hydrogenase level since the oxidation of NADH in the presence of the CoQ analog decyl-ubiquinone (DB) is lowered. Notably, Atorvastatin treatment did not affect Complex I dehydrogenase activity, since the rotenone-insensitive NADH-[Fe(CN)_6_]^3−^ reaction was not altered. In a previous study from our laboratory, Fato et al. [27] demonstrated that Complex I inhibitors can be classified into two groups based on their ability to induce reactive oxygen species (ROS). Class A inhibitors, such as rotenone, piericidin A, and rolliniastatin 1 and 2, enhance ROS production. In contrast, class B inhibitors, such as stigmatellin, mucidin, and capsaicin (that are considered classical inhibitors of Complex III at the “O” center), at high concentrations target Complex I and block it in a conformation that prevents ROS generation during forward electron transfer. Our findings suggest that the mechanism by which Atorvastatin inhibits Complex I is not rotenone-like, since Atorvastatin did not increase ROS production in isolated mitochondria energized with NADH. Since ROS could not originate from complex III—because Atorvastatin binds to the “O” center, a known site of ROS production [35]—we hypothesized that the marked increase in mitochondrial ROS observed in Atorvastatin-treated cells (Figure 8 A,B) should be attributed to reverse electron flow through complex I. To validate the occurrence of RET, we supplemented cells with succinate (a membrane-permeable metabolite) in the presence of Atorvastatin. This treatment led to an increase in ROS production, which was significantly reduced by rotenone treatment (Figure 11). Furthermore, our findings are consistent with those reported by Wojcicki et al. [36], who observed that Atorvastatin and Simvastatin increased ROS production in non-phosphorylating (State 4) mitochondria energized with succinate. In contrast, ROS levels remained low in phosphorylating (State 3) mitochondria energized with either malate-pyruvate (as NADH source) or succinate. Taken together, the results presented in this paper, along with evidence from the literature, suggest a mechanism by which Atorvastatin affects mitochondrial function. This mechanism involves the ability of the molecule to inhibit Complex III by targeting the “O” center and to alter Complex I activity. Atorvastatin inhibition may induce a Complex I conformation that prevents the direct transfer of electrons from NADH to molecular oxygen. However, in the presence of elevated levels of CoQH_2_, Atorvastatin treatment may promote reverse electron transfer (RET), leading to increased ROS production [37]. This exacerbation of oxidative stress can impair mitochondrial function and compromise cell homeostasis, apparently leaving the mitochondrial mass and the expression of genes involved in mitochondrial function unaltered.

## 5. Conclusions

Our results show that Atorvastatin impairs oxygen consumption, associated with Coenzyme Q_10_ deficiency, radical species production, and a direct inhibition of mitochondrial Complexes I and III in vitro. We propose that Atorvastatin acts as a class B inhibitor of Complex I, preventing the generation of reactive oxygen species during forward electron transport. However, ROS produced in Atorvastatin-treated HDF energized with succinate were blocked by rotenone, suggesting a reverse electron transfer.

The actual cellular concentration of the statin in vivo is not known, since impaired clearance and tissue accumulation may vary among statin users; the potential accumulation of lipophilic drugs in tissues and cells in vivo justifies using higher concentrations of statins in in vitro studies [36,38,39]. CoQ_10_ supplementation counteracts Atorvastatin-induced side effects by protecting cells from ROS and improving bioenergetic status. Further studies are needed to determine whether these findings can be extended to other statins.

## Data Availability

The datasets analyzed during the current study are available from the corresponding author upon reasonable request.

## Supplementary Materials

The following supporting information can be downloaded at: https://www.mdpi.com/article/10.3390/antiox14101147/s1, Figure S1: qPCR results; Table S1: qPCR primers; S2: uncropped DCFDA microscopy images.

## Abbreviations

The following abbreviations are used in this manuscript:

ADP	Adenosine Diphosphate
ATP	Adenosine Triphosphate
CM-H_2_DCFDA	Chloromethyl 2′-7′-dichlorodihydrofluorescein diacetate
DB	Decyl-ubiquinone
DCFDA	2′-7′-dichlorodihydrofluorescein diacetate
DCPIP	2,6-Dichlorophenolindophenol sodium salt hydrate
ETC	Electron transport chain
FCCP	Carbonyl cyanide 4-(trifluoromethoxy) Phenylhydrazone
[Fe(CN)_6_]^3−^	Ferricyanide
HBSS	Hanks’ Balanced Salt Solution
HDF	Human Dermal Fibroblasts
HMG-CoA	3-hydroxy-3-methyl-glutaryl-CoA
LDL	Low density lipoprotein
NADH	Reduced Nicotinamide adenine dinucleotide
OCR	Oxygen consumption rate
ROS	Reactive oxygen species
UBQ	UBIQSOME^®^

## References

[1] Ward N.C., Watts G.F., Eckel R.H. (2019). Statin Toxicity. Circ. Res..

[2] McFarland A.J., Anoopkumar-Dukie S., Arora D.S., Grant G.D., McDermott C.M., Perkins A.V., Davey A.K. (2014). Molecular Mechanisms Underlying the Effects of Statins in the Central Nervous System. Int. J. Mol. Sci..

[3] Goedeke L., Fernández-Hernando C. (2012). Regulation of cholesterol homeostasis. Cell Mol Life Sci..

[4] Mhaimeed O., Burney Z.A., Schott S.L., Kohli P., Marvel F.A., Martin S.S. (2024). The Importance of LDL-C Lowering in Atherosclerotic Cardiovascular Disease Prevention: Lower for Longer Is Better. Am. J. Prev. Cardiol..

[5] Zeng W., Deng H., Luo Y., Zhong S., Huang M., Tomlinson B. (2024). Advances in Statin Adverse Reactions and the Potential Mechanisms: A Systematic Review. J. Adv. Res..

[6] Laakso M., Fernandes Silva L. (2023). Statins and Risk of Type 2 Diabetes: Mechanism and Clinical Implications. Front. Endocrinol..

[7] Ezad S., Cheema H., Collins N. (2018). Statin-Induced Rhabdomyolysis: A Complication of a Commonly Overlooked Drug Interaction. Oxf. Med. Case Rep..

[8] Tricarico P.M., Crovella S., Celsi F. (2015). Mevalonate Pathway Blockade, Mitochondrial Dysfunction and Autophagy: A Possible Link. Int. J. Mol. Sci..

[9] Marcoff L., Thompson P.D. (2007). The Role of Coenzyme Q10 in Statin-Associated Myopathy: A Systematic Review. J. Am. Coll. Cardiol..

[10] Young J.M., Florkowski C.M., Molyneux S.L., McEwan R.G., Frampton C.M., George P.M., Scott R.S. (2007). Effect of Coenzyme Q10 Supplementation on *Simvastatin*-Induced Myalgia. Am. J. Cardiol..

[11] Hernández-Camacho J.D., Bernier M., López-Lluch G., Navas P. (2018). Coenzyme Q10 Supplementation in Aging and Disease. Front. Physiol..

[12] Ahmad K., Manongi N.J., Rajapandian R., Moti Wala S., Al Edani E.M., Samuel E.A., Arcia Franchini A.P. (2024). Effectiveness of Coenzyme Q10 Supplementation in Statin-Induced Myopathy: A Systematic Review. Cureus.

[13] Rizzardi N., Liparulo I., Antonelli G., Orsini F., Riva A., Bergamini C., Fato R. (2021). Coenzyme Q10 Phytosome Formulation Improves CoQ10 Bioavailability and Mitochondrial Functionality in Cultured Cells. Antioxidants.

[14] Loi M., Valenti F., Medici G., Mottolese N., Candini G., Bove A.M., Trebbi F., Pincigher L., Fato R., Bergamini C. (2025). Beneficial Antioxidant Effects of Coenzyme Q10 in In Vitro and In Vivo Models of CDKL5 Deficiency Disorder. Int. J. Mol. Sci..

[15] Jukema J.W., Cannon C.P., de Craen A.J.M., Westendorp R.G.J., Trompet S. (2012). The Controversies of Statin Therapy: Weighing the Evidence. J. Am. Coll. Cardiol..

[16] Somers T., Siddiqi S., Morshuis W.J., Russel F.G.M., Schirris T.J.J. (2023). Statins and Cardiomyocyte Metabolism, Friend or Foe?. J. Cardiovasc. Dev. Dis..

[17] Avelar-Freitas B.A., Almeida V.G., Pinto M.C.X., Mourao F.A.G., Massensini A.R., Martins-Filo O.A., Rocha-Vieira E., Brito-Melo G.E.A. (2014). Trypan blue exclusion assay by flow cytometry. Braz. J. Med. Biol. Res..

[18] Lowry O.H., Rosebrough N.J., Farr A.L., Randall R.J. (1951). Protein Measurement with the Folin Phenol Reagent. J. Biol. Chem..

[19] van Meerloo J., Kaspers G.J.L., Cloos J. (2011). Cell Sensitivity Assays: The MTT Assay. Methods Mol. Biol. Clifton NJ.

[20] Jones D.P. (1981). Determination of Pyridine Dinucleotides in Cell Extracts by High-Performance Liquid Chromatography. J. Chromatogr. B. Biomed. Sci. App..

[21] Takada M., Ikenoya S., Yuzuriha T., Katayama K. (1984). [17] Simultaneous Determination of Reduced and Oxidized Ubiquinones. Methods in Enzymology.

[22] Larsen S., Nielsen J., Hansen C.N., Nielsen L.B., Wibrand F., Stride N., Schroder H.D., Boushel R., Helge J.W., Dela F. (2012). Biomarkers of Mitochondrial Content in Skeletal Muscle of Healthy Young Human Subjects. J. Physiol..

[23] Bergamini C., Fato R., Biagini G., Pugnaloni A., Giantomassi F., Foresti E., Lesci G.I., Roveri N., Lenaz G. (2007). Mitochondrial Changes Induced by Natural and Synthetic Asbestos Fibers: Studies on Isolated Mitochondria. Cell. Mol. Biol. Noisy—Gd. Fr..

[24] Spinazzi M., Casarin A., Pertegato V., Salviati L., Angelini C. (2012). Assessment of Mitochondrial Respiratory Chain Enzymatic Activities on Tissues and Cultured Cells. Nat. Protoc..

[25] Askerlund P., Larsson C., Widell S. (1988). Localization of Donor and Acceptor Sites of NADH Dehydrogenase Activities Using inside-Out and Right-Side-Out Plasma Membrane Vesicles from Plants. FEBS Lett..

[26] Pedrera L., Prieto Clemente L., Dahlhaus A., Lotfipour Nasudivar S., Tishina S., Olmo González D., Stroh J., Yapici F.I., Singh R.P., Grotehans N. (2025). Ferroptosis Triggers Mitochondrial Fragmentation via Drp1 Activation. Cell Death Dis..

[27] Fato R., Bergamini C., Bortolus M., Maniero A.L., Leoni S., Ohnishi T., Lenaz G. (2009). Differential Effects of Mitochondrial Complex I Inhibitors on Production of Reactive Oxygen Species. Biochim. Biophys. Acta BBA—Bioenerg..

[28] Mach F., Baigent C., Catapano A.L., Koskinas K.C., Casula M., Badimon L., Chapman M.J., De Backer G.G., Delgado V., Ference B.A. (2020). 2019 ESC/EAS Guidelines for the Management of Dyslipidaemias: Lipid Modification to Reduce Cardiovascular Risk: The Task Force for the Management of Dyslipidaemias of the European Society of Cardiology (ESC) and European Atherosclerosis Society (EAS). Eur. Heart J..

[29] Shi Z., Han S. (2025). Personalized Statin Therapy: Targeting Metabolic Processes to Modulate the Therapeutic and Adverse Effects of Statins. Heliyon.

[30] Gbelcová H., Rimpelová S., Jariabková A., Macášek P., Priščáková P., Ruml T., Šáchová J., Kubovčiak J., Kolář M., Vítek L. (2024). Highly Variable Biological Effects of Statins on Cancer, Non-Cancer, and Stem Cells in Vitro. Sci. Rep..

[31] Ahmadi Y., Fard J.K., Ghafoor D., Eid A.H., Sahebkar A. (2023). Paradoxical Effects of Statins on Endothelial and Cancer Cells: The Impact of Concentrations. Cancer Cell Int..

[32] Khatiwada N., Hong Z. (2024). Potential Benefits and Risks Associated with the Use of Statins. Pharmaceutics.

[33] Friedrich T., Dekovic D.K., Burschel S. (2016). Assembly of the *Escherichia coli* NADH:Ubiquinone Oxidoreductase (Respiratory Complex I). Biochim. Biophys. Acta BBA—Bioenerg..

[34] Brandt U. (2006). Energy Converting NADH:Quinone Oxidoreductase (Complex I). Annu. Rev. Biochem..

[35] Schirris T.J.J., Renkema G.H., Ritschel T., Voermans N.C., Bilos A., van Engelen B.G.M., Brandt U., Koopman W.J.H., Beyrath J.D., Rodenburg R.J. (2015). Statin-Induced Myopathy Is Associated with Mitochondrial Complex III Inhibition. Cell Metab..

[36] Wojcicki K., Budzinska A., Jarmuszkiewicz W. (2024). Effects of Atorvastatin and Simvastatin on the Bioenergetic Function of Isolated Rat Brain Mitochondria. Int. J. Mol. Sci..

[37] Goncalves R.L.S., Wang Z.B., Riveros J.K., Parlakgül G., Inouye K.E., Lee G.Y., Fu X., Saksi J., Rosique C., Hui S.T. (2025). CoQ Imbalance Drives Reverse Electron Transport to Disrupt Liver Metabolism. Nature.

[38] Cilla D.D., Whitfield L.R., Gibson D.M., Sedman A.J., Posvar E.L. (1996). Multiple-Dose Pharmacokinetics, Pharmacodynamics, and Safety of Atorvastatin, an Inhibitor of HMG-CoA Reductase, in Healthy Subjects. Clin. Pharmacol. Ther..

[39] Björkhem-Bergman L., Lindh J.D., Bergman P. (2011). What Is a Relevant Statin Concentration in Cell Experiments Claiming Pleiotropic Effects?. Br. J. Clin. Pharmacol..

