# Supplementary material for: Atorvastatin Induces Bioenergetic Impairment and Oxidative Stress Through Reverse Electron Transport
Source: Antioxidants (Basel). 2025 Sep 23;14(10):1147. doi: 10.3390/antiox14101147 (PMC12561091; doi:10.3390/antiox14101147)
Supplement: Supplementary file 1 [file antioxidants-14-01147-s001.zip › Supplementary-antioxidants-3760031_qPCR.pdf]

Supplementary material.

Real Time PCR

A set of genes central for mitochondrial function was tested with Real-Time PCR. Briefly, 2×10^5 cells were seeded in a multi-6 well plate and allowed to attach for 24 hours before treatment. Cells were incubated with 30 μM of Atorvastatin with and without 10 nM of UBQ for 6 hours. After the incubation period, cells were washed with cold PBS, detached by trypsin, and centrifuged at 7500 rpm at 4°C. The pellets were transferred into liquid nitrogen and stored at -80°C until processed. Gene expression analysis was used to quantify the possible change in expression of a selected set of genes: citrate synthase (CS), succinate dehydrogenase (SDH), peroxisome proliferator-activated receptor gamma coactivator 1-alpha (PGC-1α), and electron-transferring-flavoprotein dehydrogenase (ETFDH). SYBR-green Real-Time qPCR (PowerUp™ SYBR™ Green Master Mix; ThermoFisher) was used for gene expression analysis, using β-Actin and GAPDH as housekeeping genes. QPCR experiments were run in triplicate in 3 biological replicates. The 2-ΔΔCt method was used for relative gene expression quantification. Primer sequences are listed in Supplementary Table 1.

Supplementary Table 1 – Primer sequences for Real-Time qPCR

Gene	Gene accession number	Primer sequences
**β-ACTIN**	NM001101	Fw 5′-ACCTTCTACAATGAGCTGCG-3′<br>Rv 5′-CCTGGATAGCAACGTACATGG-3′
**GAPDH**	NM_002046	Fw 5′-ACATCGCTCAGACACCATG-3′<br>Rv 5′-TGTAGTTGAGGTCAATGAAGGG-3′
**CS**	NM_004077.3	Fw 5′-CATTGACTCTAACCTGGACTGG-3′<br>Rv 5′-ACTTACATTGCCACCCTCATG-3′
**SDH**	NM_004168.2	Fw 5′-TGGTTGTCTTTGGTCGGG-3′<br>Rv 5′-GCGTTTGGTTTAATTGGAGGG-3′
**PGC1α**	NM_013261.5	Fw 5′-ACCAAACCCACAGAGAACAG-3′<br>Rv 5′-GGGTCAGAGGAAGAGATAAAGTTG-3′
**ETFDH**	NM_004453.4	Fw 5′-TTCAACTTCTACTGTGCCTCG-3′<br>Rv 5′-GCCTGCACCAACTATTACAAC-3′

Results

QPCR analysis revealed no significant change in the expression of the selected genes, regardless of the reference gene used (Figure S1 A, B).

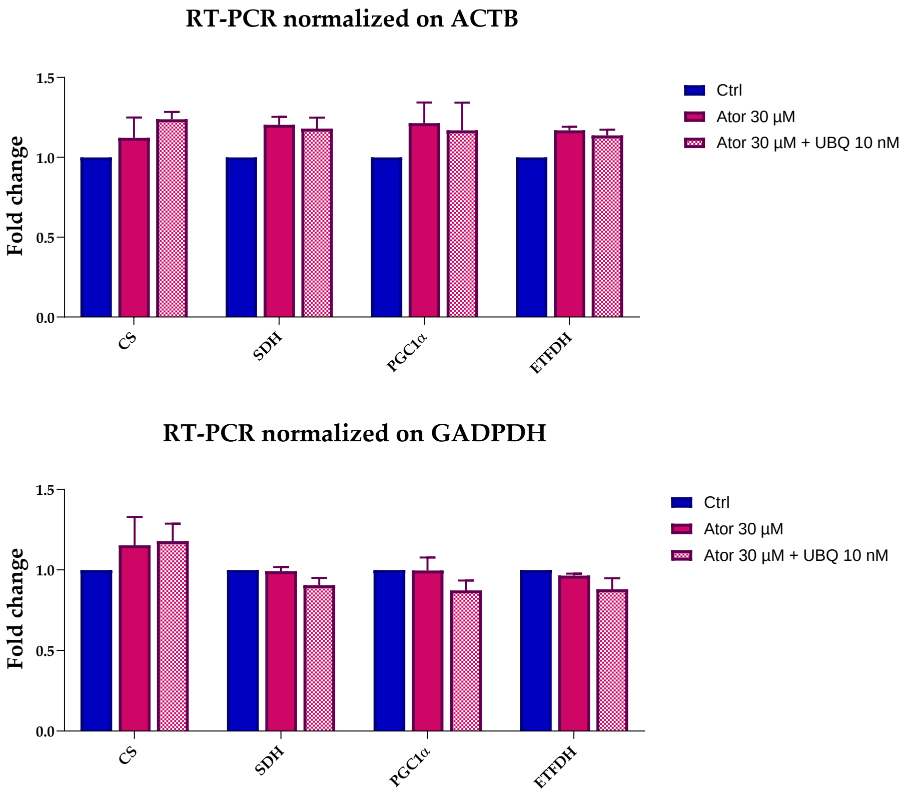

Figure S1. Real Time PCR analysis in human dermal fibroblasts following a 6-hour treatment with 30 µM of Atorvastatin alone or in combination with 10 nM UBIQSOME®. The Y-axis reports 2-ΔΔCt values corresponding to gene expression normalized to controls. Data represent relative gene expressions of citrate synthase, succinate dehydrogenase, peroxisome proliferator-activated receptor gamma coactivator 1-alpha, and electron-transferring-flavoprotein dehydrogenase, normalized on β-Actin (A) and GAPDH (B). Data are expressed as mean ± SEM (n = 3). Statistical significance was assessed using Tukey's multiple comparisons test. Abbreviations: Ator = Atorvastatin; UBQ = UBIQSOME®; CS = citrate synthase; SDH = succinate dehydrogenase; PGC-1α = peroxisome proliferator-activated receptor gamma coactivator 1-alpha; ETFDH = electron-transferring-flavoprotein dehydrogenase.
