# Supplementary figures and images for: Atorvastatin Induces Bioenergetic Impairment and Oxidative Stress Through Reverse Electron Transport
Source: Antioxidants (Basel). 2025 Sep 23;14(10):1147. doi: 10.3390/antiox14101147 (PMC12561091; doi:10.3390/antiox14101147)

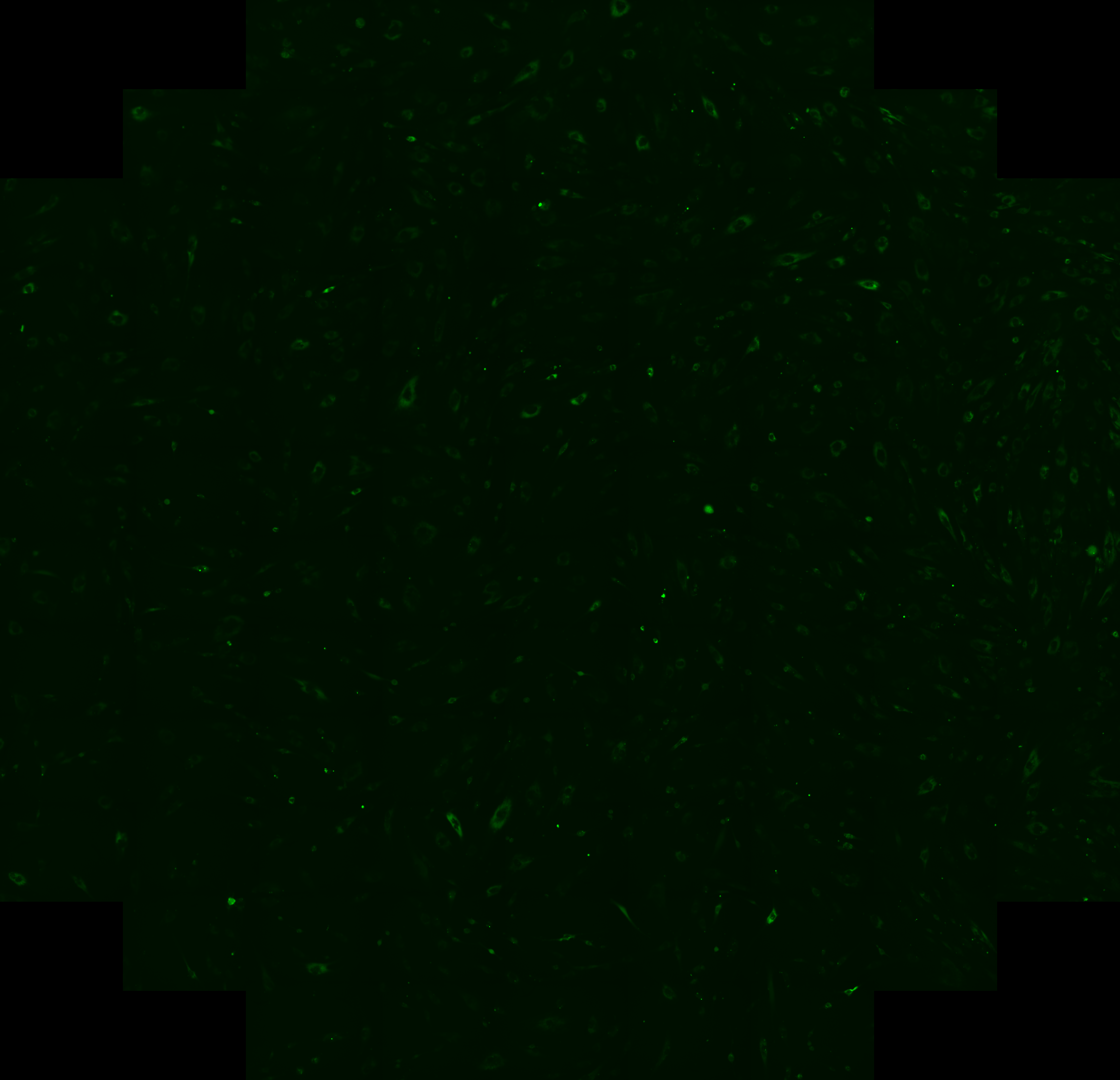

Supplement: Supplementary file 1 [file antioxidants-14-01147-s001.zip › DCFDA 20x-01 ctrl.png]

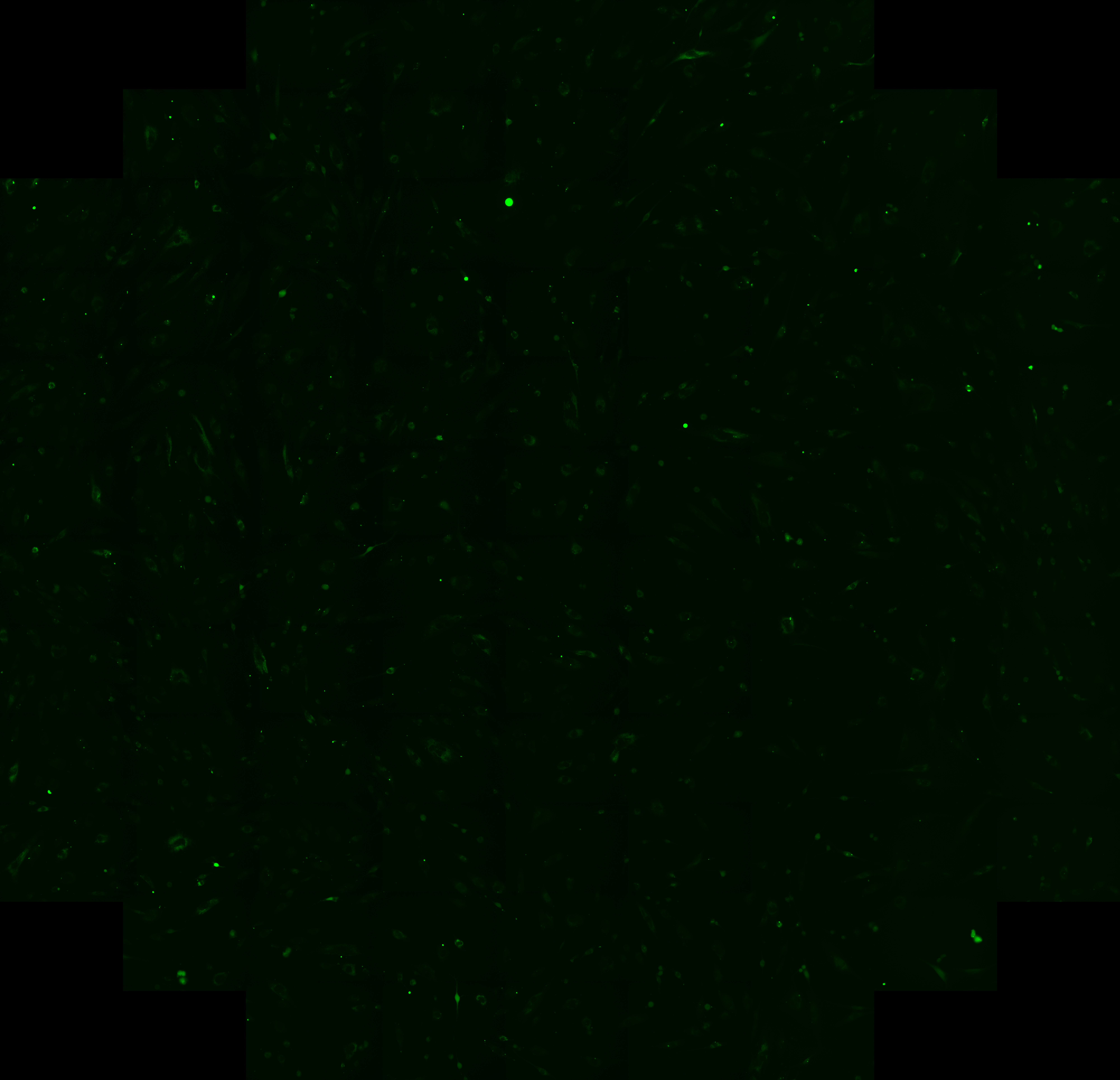

Supplement: Supplementary file 1 [file antioxidants-14-01147-s001.zip › DCFDA 20x-01 UBQ.png]

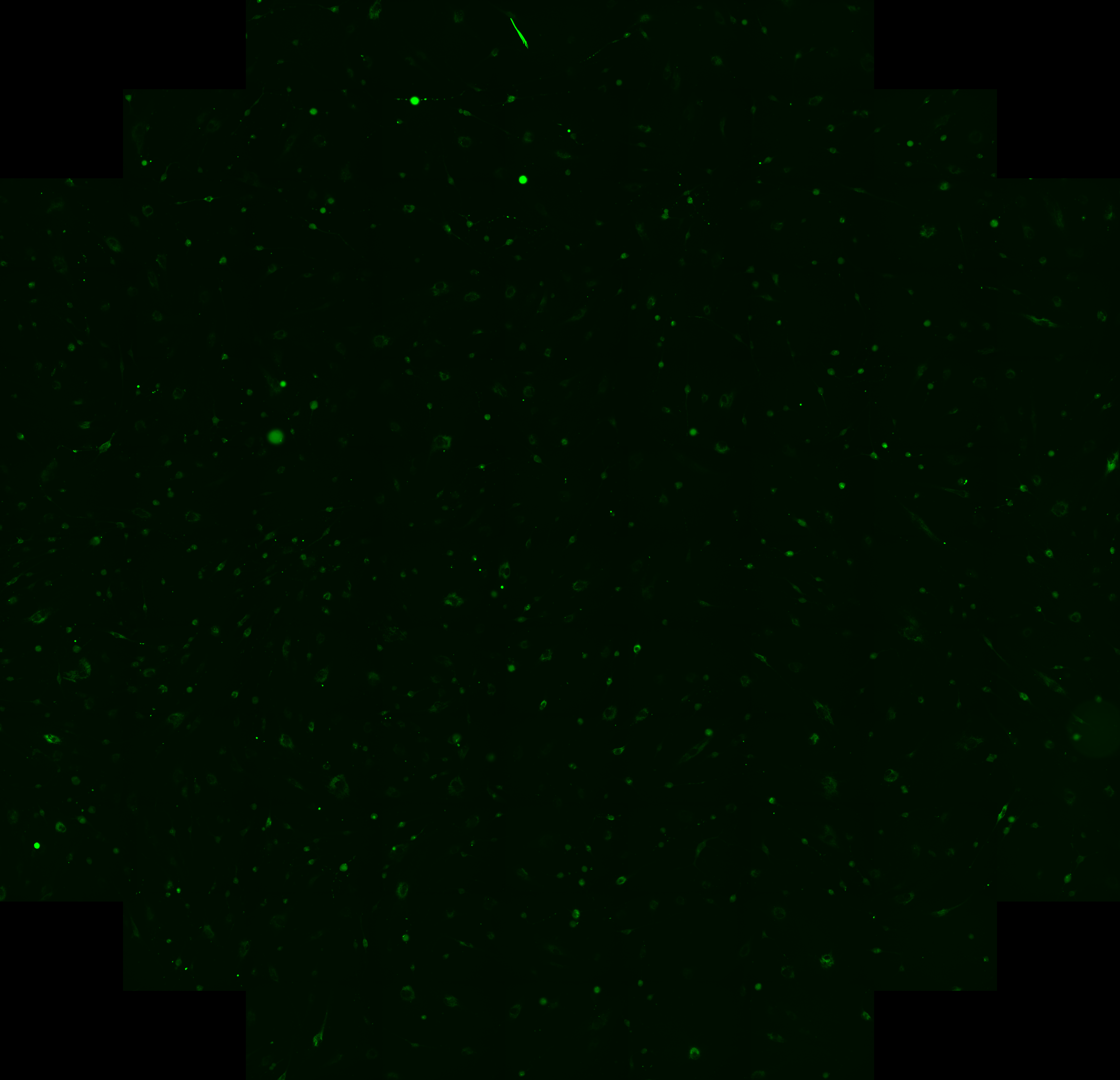

Supplement: Supplementary file 1 [file antioxidants-14-01147-s001.zip › DCFDA 20x-01-ATOR UBQ.png]

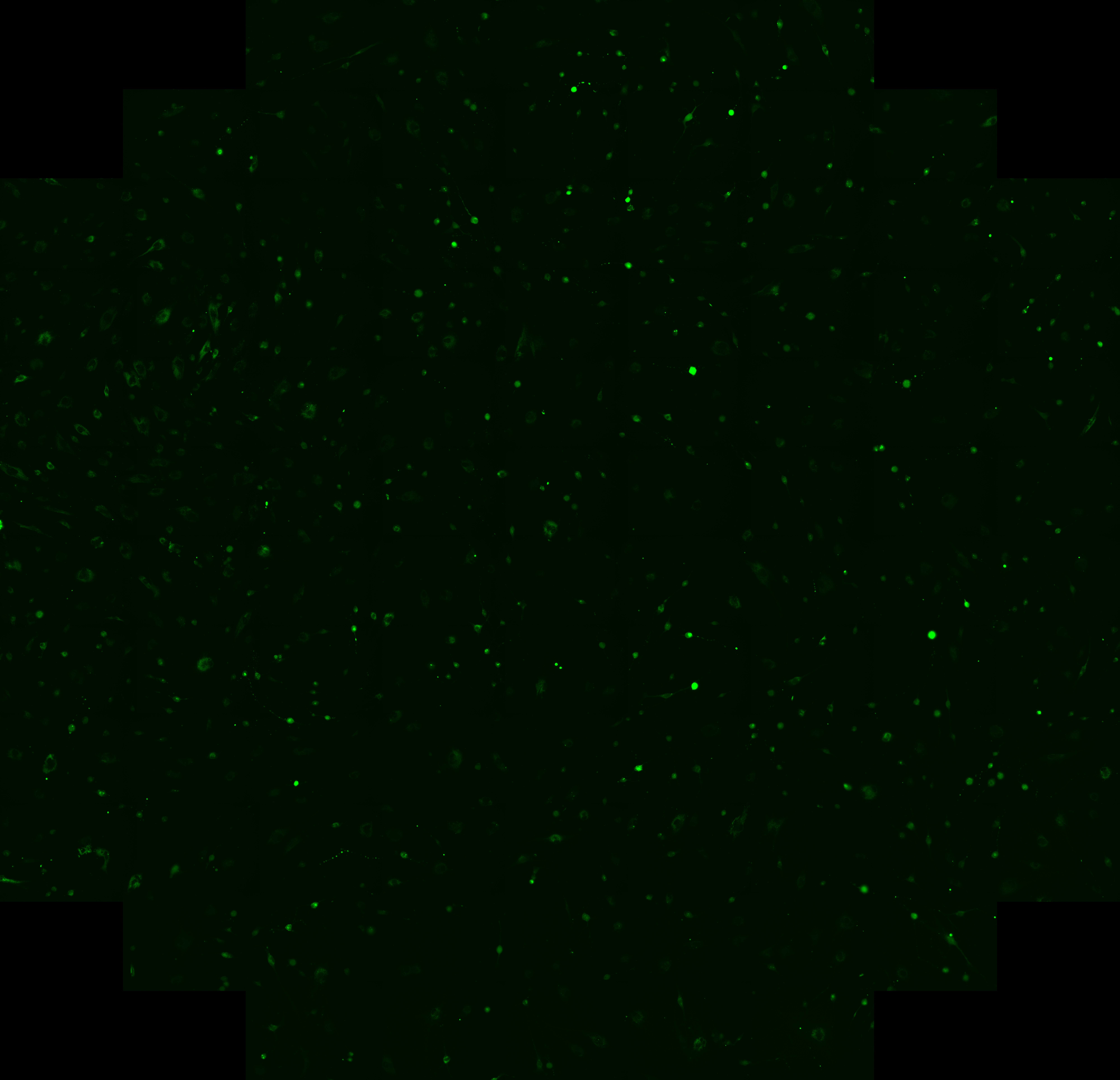

Supplement: Supplementary file 1 [file antioxidants-14-01147-s001.zip › DCFDA 20x-01-ATOR.png]

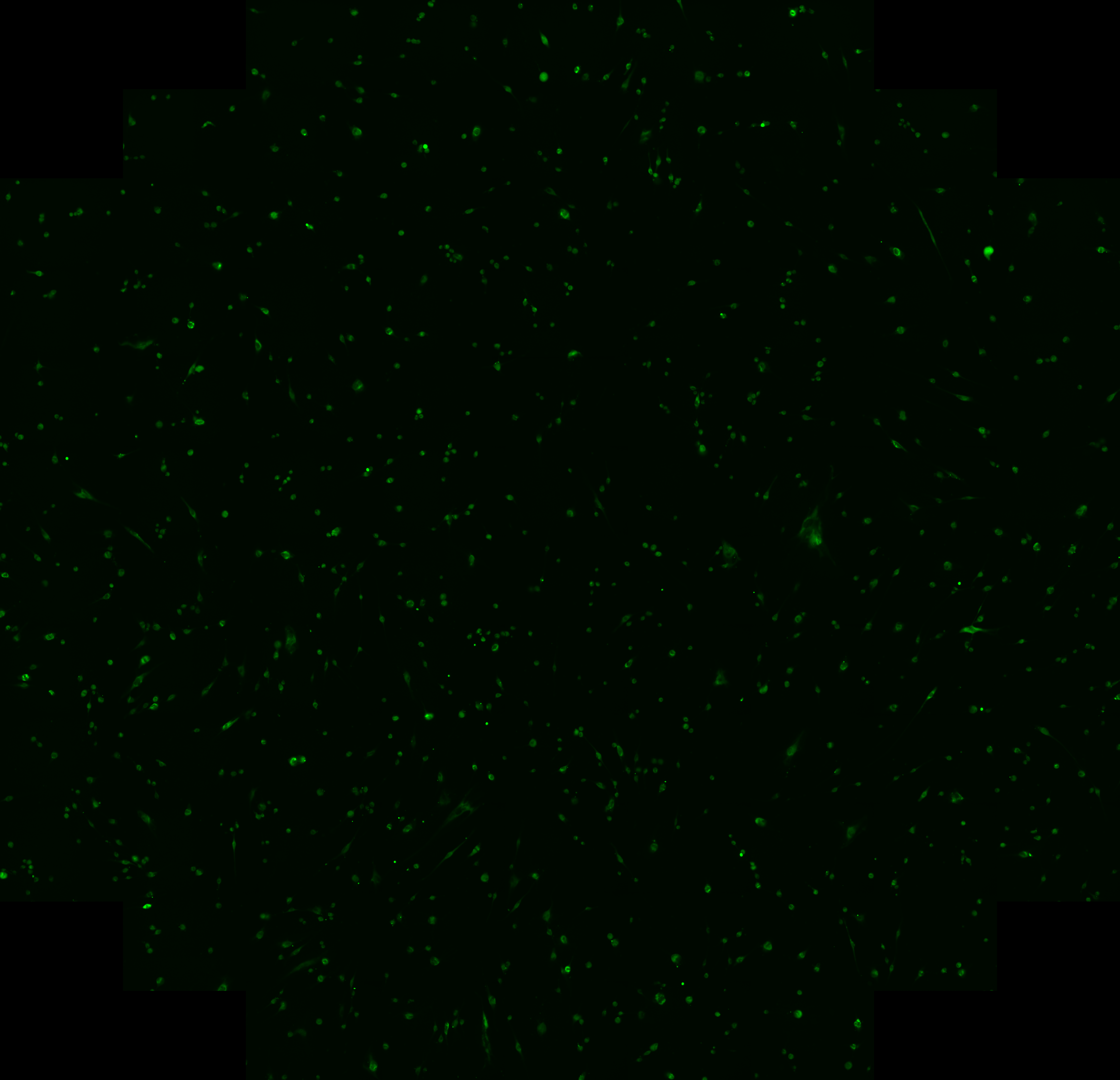

Supplement: Supplementary file 1 [file antioxidants-14-01147-s001.zip › DCFDA PEROXIDO 20x positive ctrl ubq.png]

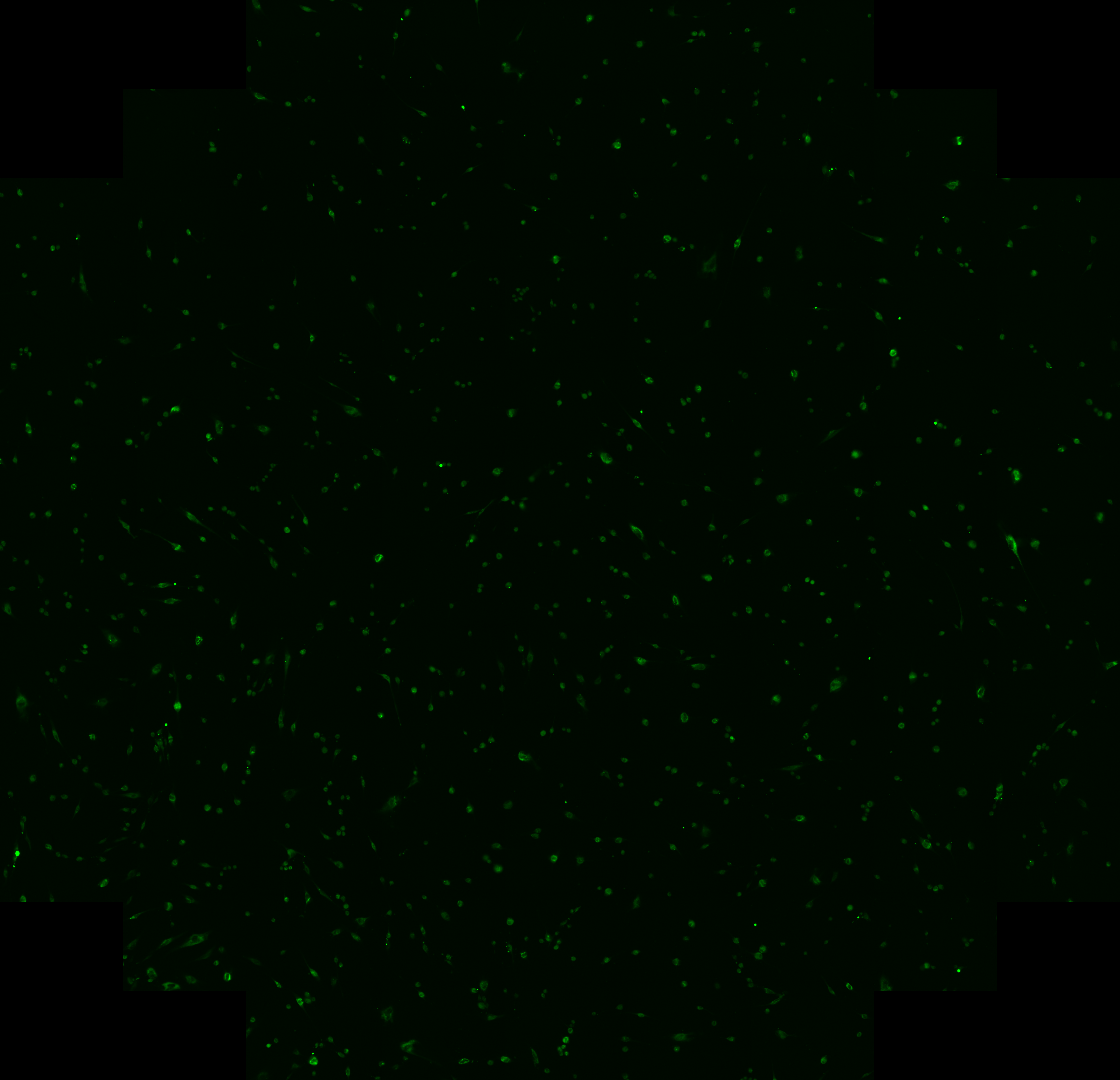

Supplement: Supplementary file 1 [file antioxidants-14-01147-s001.zip › DCFDA PEROXIDO 20x- positive ctrl.png]
